# Supplementary material for: Anti-nucleocapsid and anti-spike antibody trajectories in people with post-covid condition versus acute-only infections: a nested longitudinal case-control study within the Virus Watch prospective cohort
Source: Nat Commun. 2025 Apr 17;16:3686. doi: 10.1038/s41467-025-58766-7 (PMC12006544; doi:10.1038/s41467-025-58766-7)
Supplement: Supplementary file 1 — Supplementary Information [file 41467_2025_58766_MOESM1_ESM.pdf]

Supplementary Materials

Supplementary Figure 1. Schematic Diagram of Virus Watch Data Collection and Post-Covid Condition Classification

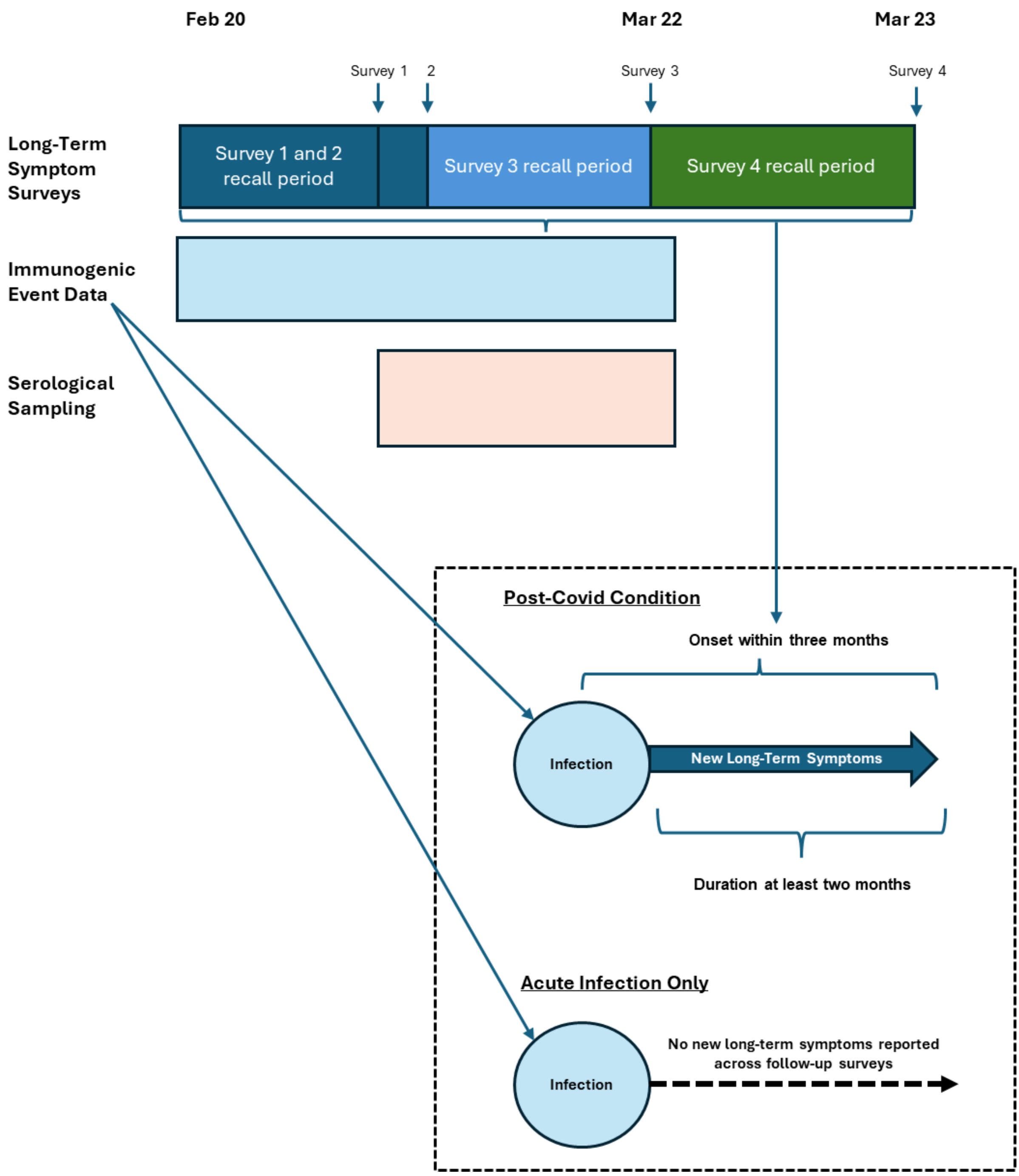

Supplementary Figure 2. Schematic Diagram of Antibody Trajectory Models for Anti-Nucleocapsid (a) and Anti-Spike (b) Antibodies

a. **Anti-Nucleocapsid Model**

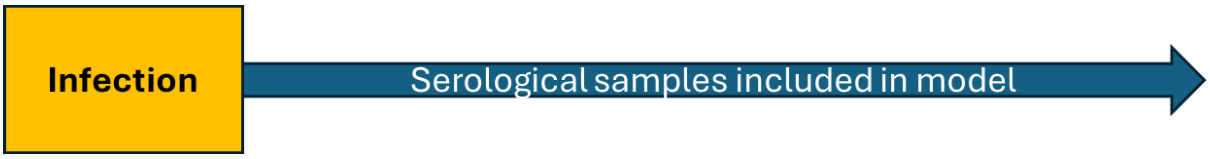

b. **Anti-Spike Models** (Pre-Infection Vaccination-Only and Post-Infection Hybrid Immunity Models)

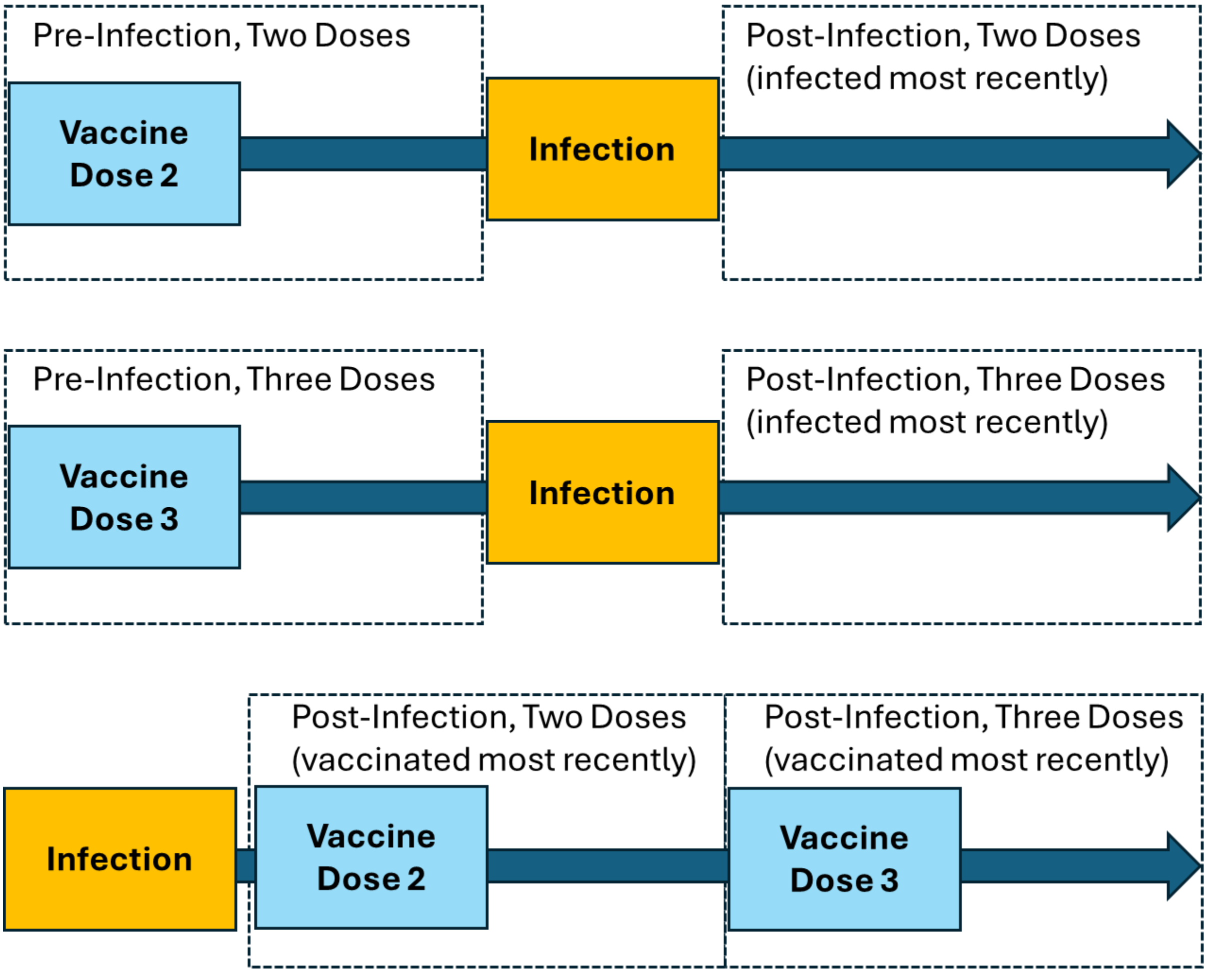

Note: The anti-Spike diagram above (Panel B) intends to provide examples of the timing of relevant immunogenic events (vaccination and/or infection) within each model. The distance between these events can vary, and events may be combined beyond the illustration above, which was simplified for clarity (e.g., dose two of vaccination may occur prior to infection and dose three afterwards, etc.).

Supplementary Figure 3a. Conceptual Model of Analysis: Post-Infection Anti-Nucleocapsid Antibody Response

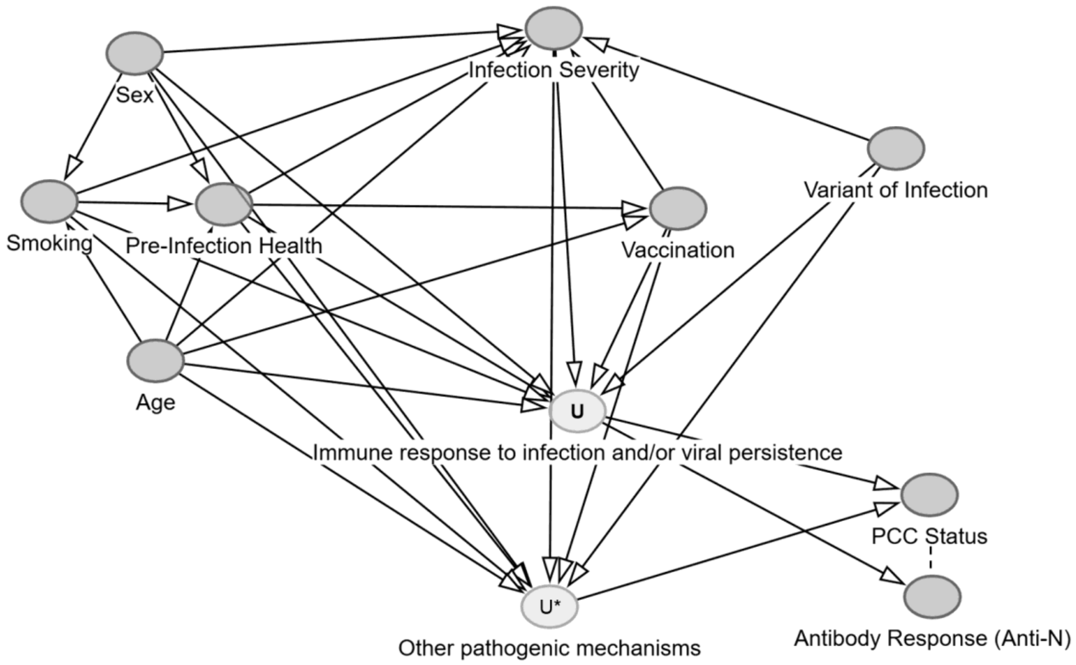

Note: U* is included for illustrative purposes; U denotes is the unmeasured mechanism underlying the analyses

Supplementary Figure 3b. Conceptual Model of Analysis: Post-Infection Anti-Spike Antibody Response

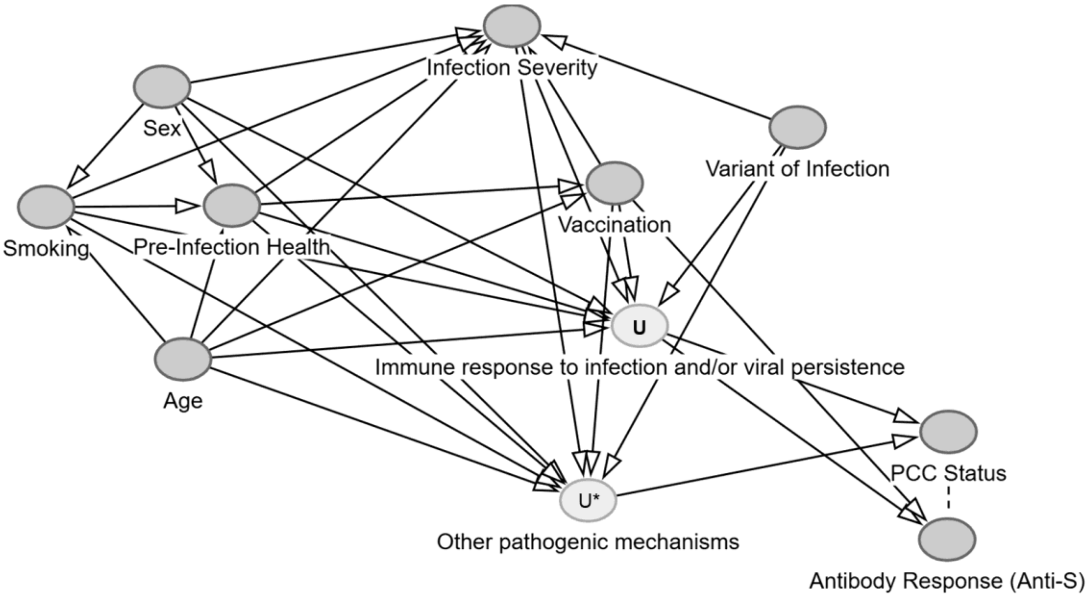

Note: U* is included for illustrative purposes; U denotes is the unmeasured mechanism underlying the analyses

Supplementary Figure 3c. Conceptual Model of Analysis: Post-Vaccination (Pre-Infection) Anti-Spike Antibody Response

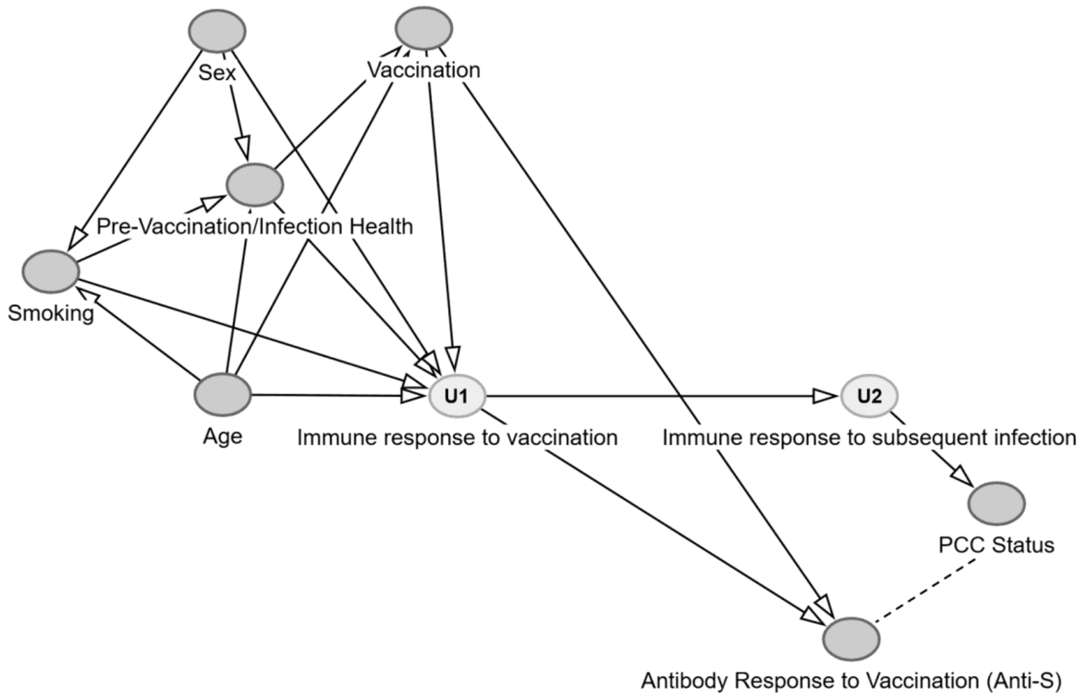

Supplementary Figure 4. Flowchart of Participant Selection

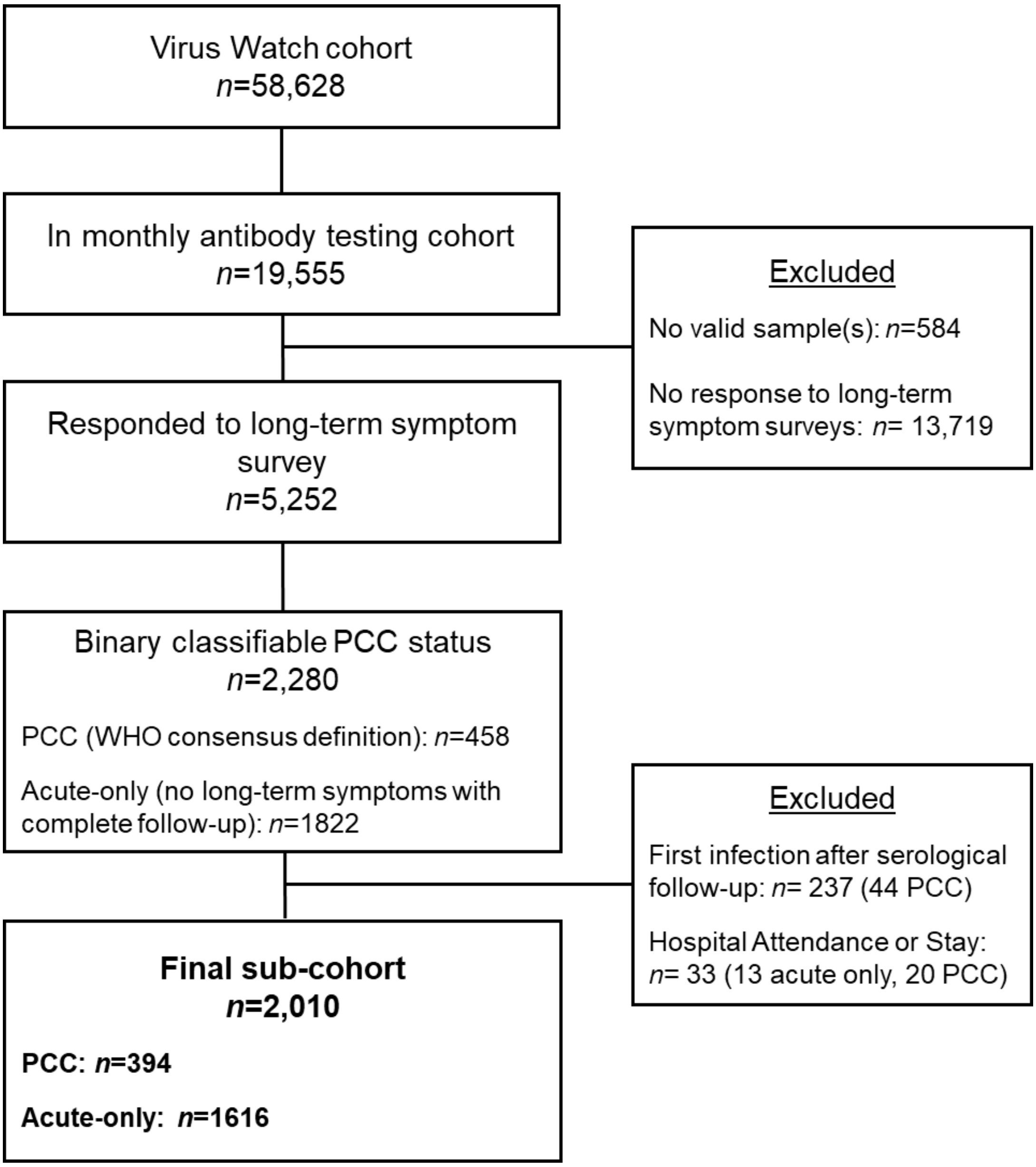

Note: PCC=Post-Covid Condition

Supplementary Table 1. Number of Samples per Antibody Trajectory Model

**Model**	**Overall (*n*)****	**Acute only (*n*)**	**PCC (*n*)**
Anti-N	1899	1229	670
Anti-N (seropositivity sensitivity analysis)	1411	835	576
Anti-S (pre-infection, two doses)	5461	4775	686
Anti-S (pre-infection, three doses)	2051	1790	261
Anti-S (post-infection, two doses - vaccinated most recently)	455	241	214
Anti-S (post-infection, two doses - infected most recently)	93	73	20
Anti-S (post-infection, three doses - vaccinated most recently)	356	299	57
Anti-S (post-infection, three doses - infected most recently)	186	133	53

PCC=Post-Covid Condition
**Note: the sum of samples across models exceeds the total number of samples in the study (*n*=9466) due to testing for both S and N antibodies leading to inclusion of samples in multiple models.

Supplementary Table 2. Frequencies and Odds of Detectable Anti-N Seropositivity in People with PCC versus Acute Infection Only, by Time Since Infection

Group	Total (Acute Only)	Seropositive (Acute Only)	Total (PCC)	Seropositive (PCC)	OR	95% CI	p-value
Overall	410	309 (75%)	190	161 (85%)	1.81	1.16, 2.90	0.010
0-29 Days	240	134 (56%)	59	37 (63%)	1.33	0.75, 2.42	0.3
30-59 Days	184	144 (78%)	54	46 (85%)	1.60	0.73, 3.90	0.3
60-89 Days	156	116 (74%)	68	59 (87%)	2.26	1.07, 5.25	0.043
90-119 Days	112	76 (68%)	64	57 (89%)	3.86	1.69, 10.0	0.003
120-269 Days	148	94 (64%)	100	87 (87%)	3.84	2.02, 7.80	<0.001
270+ Days	69	35 (51%)	67	55 (82%)	4.45	2.08, 10.0	<0.001

PCC=Post-Covid Condition; OR = odds ratio; CI = confidence interval; odds ratios and 95% CIs obtained from binary logistic regression

Supplementary Table 3. Odds Ratios and Likelihood Ratio Tests for Interaction between Sex and Comorbidity Status and PCC Status on Anti-N Seropositivity (Overall Model Across Full Follow-Up Period)

Note: In the presence of an interaction term, main effects cannot be interpreted directly. For the main effect of PCC status, please see Supplementary Table 2 and Figure 2. Main effects of sex and comorbidities on anti-N seroconversion have been investigated elsewhere in the Virus Watch data[1] using appropriate methodology and samples.

**Group**	**Characteristic**	**OR[1]**	**95% CI**	***p*-value**	**LRT *p***
Sex	PCC Status (see note above)	1.81	1.09, 3.10	0.026	
	Sex (see note above)	1.58	0.99, 2.55	0.059	
	**PCC Status * Sex**	**1.33**	**0.46, 4.50**	**0.6**	**0.05**
Comorbidities	PCC Status (see note above)	2.37	1.20, 5.12	0.018	
	Comorbidities (see note above)	0.76	0.48, 1.22	0.3	
	**PCC Status * Comorbidities**	**0.70**	**0.26, 1.77**	**0.5**	**0.16**
Vaccination Status	PCC Status (see note above)	3.12	1.48, 6.81	0.003	

	Vaccination Status (see note above)	1.34	0.81, 2.22	0.30	
	**PCC Status * Vaccination Status[2]**	**0.53**	**0.30, 0.97**	**0.04**	**0.07**
Variant	PCC Status (see note above)	2.98	1.59, 5.82	<0.001	
	Variant (see note above)	1.72	0.80, 3.64	0.02	
	**PCC Status * Variant**	**0.21**	**0.08, 0.57**	**0.003**	**0.01**

Note: OR = odds ratio; 95% CI = 95% confidence interval; LRT = likelihood ratio test; PCC = Post-Covid Condition; odds ratios and 95% CIs obtained from binary logistic regression and likelihood ratio tests performed for interaction term of associated logistic regressions

[1] Navaratnam AM, Shrotri M, Nguyen V, Braithwaite I, Beale S, Byrne TE, Fong WL, Fragaszy E, Geismar C, Hoskins S, Kovar J. Nucleocapsid and spike antibody responses following virologically confirmed SARS-CoV-2 infection: an observational analysis in the Virus Watch community cohort. International Journal of Infectious Diseases. 2022 Oct 1;123:104-11.

[2]Likelihood ratio test conducted on subset of participants who had 0, 2, or 3 dose vaccination due to small subgroup sizes for 1 dose or 4+ doses precluding inclusion in analyses

Supplementary Figure 5. Anti-Nucleocapsid Antibody Trajectories in Participants with Post-COVID Condition versus Acute Infection Only: Sensitivity analysis limited to anti-N seroconverters

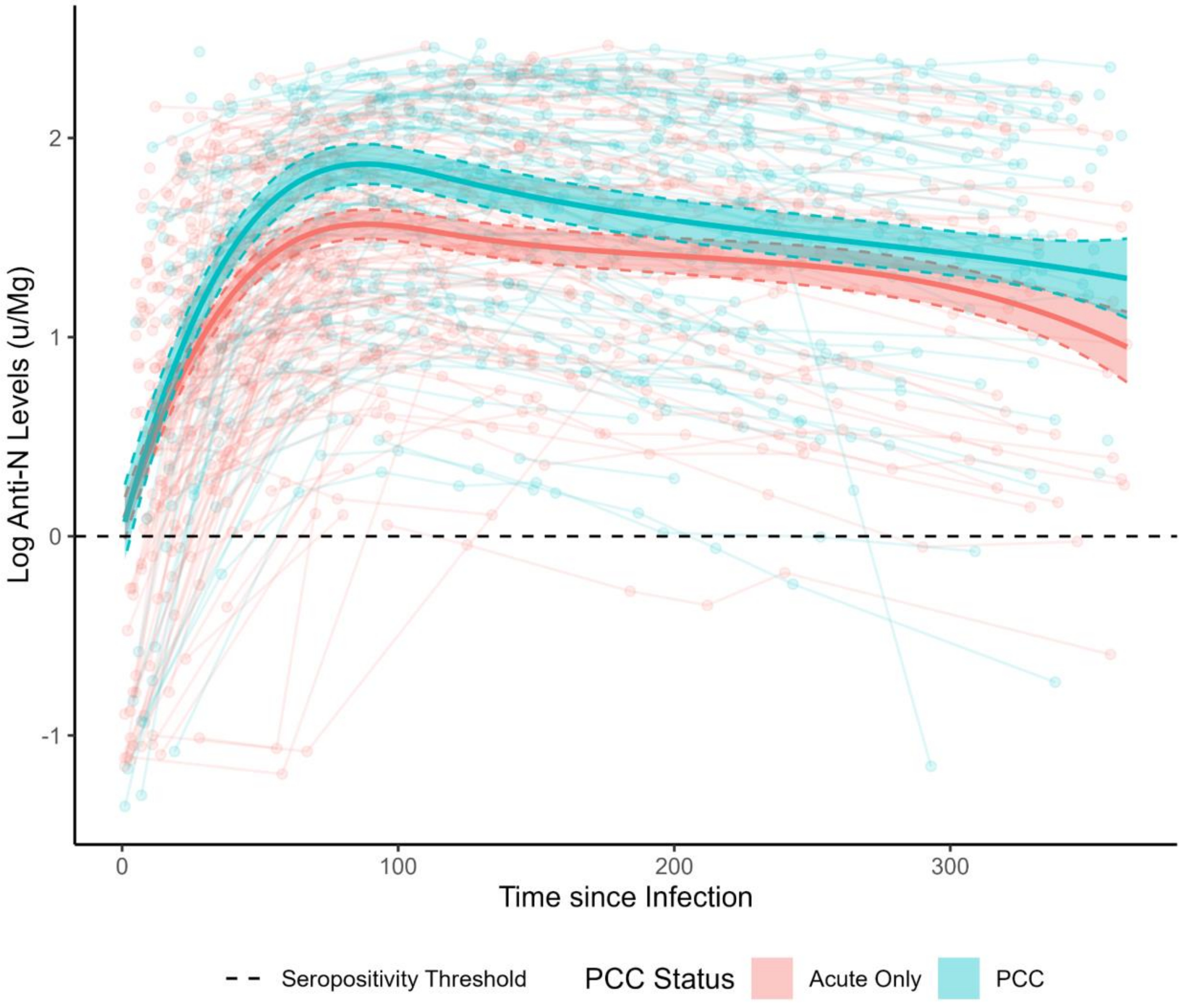

Note: *n*=1422 samples from 475 participants within this sensitivity analysis; the legend applies to all panels in the figure.

Supplementary Figure 6. Post-Infection, Post-Vaccination Anti-Spike Antibody Trajectories in Participants with Post-COVID Condition versus Acute Infection Only: Post-two-dose vaccination with vaccination (a) and infection (b) most recently and three-dose vaccination with vaccination (c) and infection (d) most recently

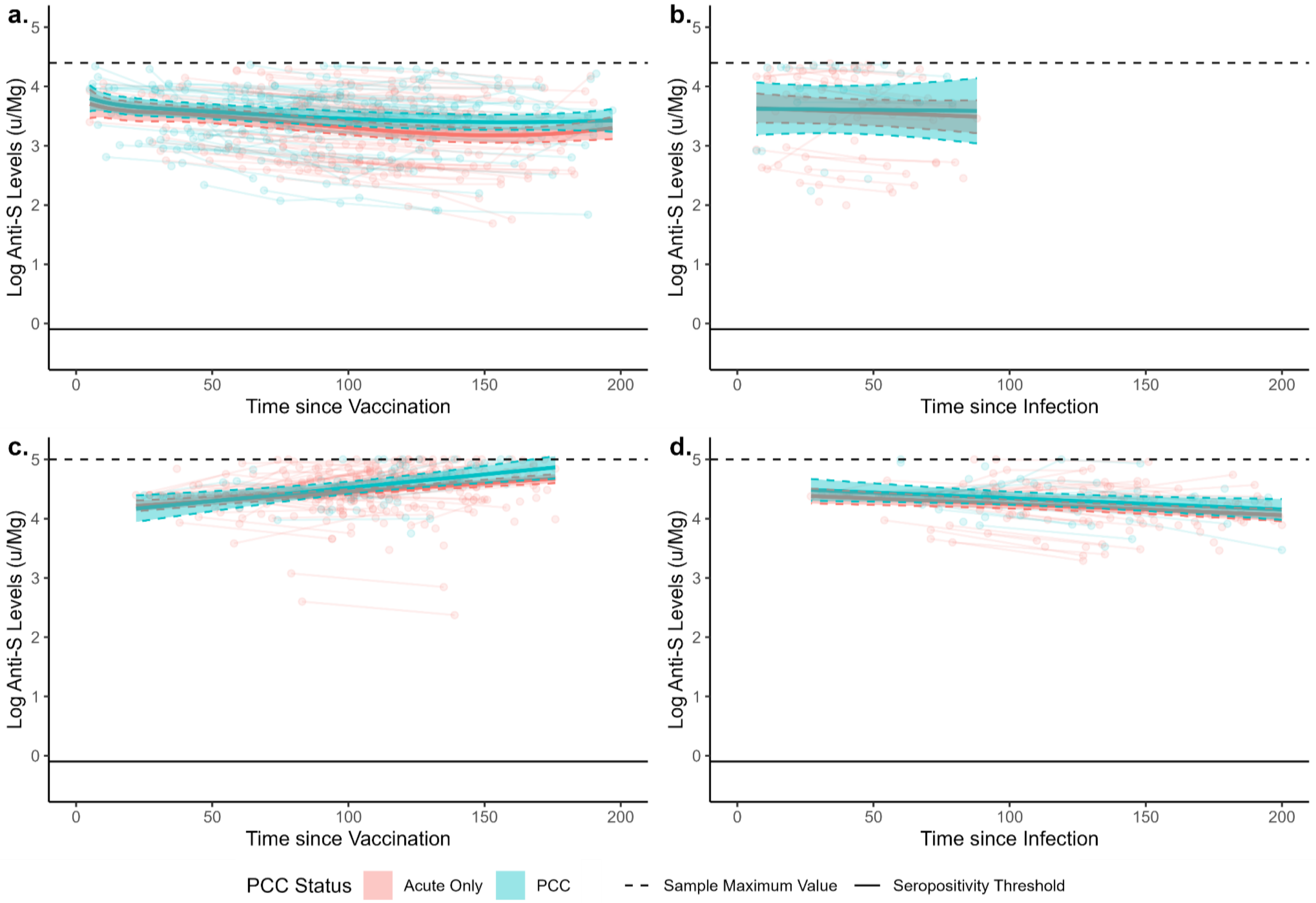

Note: The legend applies to all panels in the figure.
