# Supplementary material for: Anti-nucleocapsid and anti-spike antibody trajectories in people with post-covid condition versus acute-only infections: a nested longitudinal case-control study within the Virus Watch prospective cohort
Source: Nat Commun. 2025 Apr 17;16:3686. doi: 10.1038/s41467-025-58766-7 (PMC12006544; doi:10.1038/s41467-025-58766-7)
Supplement: Supplementary file 3 — Reporting Summary [file 41467_2025_58766_MOESM3_ESM.pdf]

nature portfolio

Corresponding author(s): Sarah Beale

Last updated by author(s): Mar 14, 2025

Reporting Summary

Nature Portfolio wishes to improve the reproducibility of the work that we publish. This form provides structure for consistency and transparency in reporting. For further information on Nature Portfolio policies, see our Editorial Policies and the Editorial Policy Checklist.

Statistics

For all statistical analyses, confirm that the following items are present in the figure legend, table legend, main text, or Methods section.

n/a	Confirmed	
☐	☒	The exact sample size (*n*) for each experimental group/condition, given as a discrete number and unit of measurement
☐	☒	A statement on whether measurements were taken from distinct samples or whether the same sample was measured repeatedly
☐	☒	The statistical test(s) used AND whether they are one- or two-sided<br>*Only common tests should be described solely by name; describe more complex techniques in the Methods section.*
☐	☒	A description of all covariates tested
☐	☒	A description of any assumptions or corrections, such as tests of normality and adjustment for multiple comparisons
☐	☒	A full description of the statistical parameters including central tendency (e.g. means) or other basic estimates (e.g. regression coefficient) AND variation (e.g. standard deviation) or associated estimates of uncertainty (e.g. confidence intervals)
☐	☒	For null hypothesis testing, the test statistic (e.g. *F*, *t*, *r*) with confidence intervals, effect sizes, degrees of freedom and *P* value noted<br>*Give P values as exact values whenever suitable.*
☒	☐	For Bayesian analysis, information on the choice of priors and Markov chain Monte Carlo settings
☐	☒	For hierarchical and complex designs, identification of the appropriate level for tests and full reporting of outcomes
☒	☐	Estimates of effect sizes (e.g. Cohen's *d*, Pearson's *r*), indicating how they were calculated

Our web collection on statistics for biologists contains articles on many of the points above.

Software and code

Policy information about availability of computer code

Data collection	Described in detail in the Method section, software not involved
Data analysis	Data analysis: Described in detail in the Methods section.<br>Code availability: We have provided de-identified example code which adhere to the principles of data protection required by the project at the following link - https://doi.org/10.17605/OSF.IO/TA2BU.

For manuscripts utilizing custom algorithms or software that are central to the research but not yet described in published literature, software must be made available to editors and reviewers. We strongly encourage code deposition in a community repository (e.g. GitHub). See the Nature Portfolio guidelines for submitting code & software for further information.

Data

Policy information about availability of data

All manuscripts must include a data availability statement. This statement should provide the following information, where applicable:

- Accession codes, unique identifiers, or web links for publicly available datasets
- A description of any restrictions on data availability
- For clinical datasets or third party data, please ensure that the statement adheres to our policy

Individual record serological data used in this study have been deposited in the Office of National Statistics Secure Research Service (ONS SRS) database under accession code 89201 https://ons.metadata.works/browser/dataset/89201. The Virus Watch study data are available under restricted access due to ethical and

legal restrictions around individual-level health-related sensitive data, access can be obtained by following the procedure described in the ONS SRS website link about. The raw Virus Watch data are protected and are not available due to data privacy laws.

Research involving human participants, their data, or biological material

Policy information about studies with human participants or human data. See also policy information about sex, gender (identity/presentation), and sexual orientation and race, ethnicity and racism.

Reporting on sex and gender	Yes, this information has been collected, is described using the recommended terms, and is presented for the data along with disaggregated analyses as requested within the Methods and Results sections
Reporting on race, ethnicity, or other socially relevant groupings	N/A
Population characteristics	The 2010 participants had a median age of 64 (interquartile range 57, 70), and comprised 1,280 (59%) people reporting female sex at birth and 888 (41%) reporting male sex at birth, of whom 802 (40%) had a preexisting condition rendering them vulnerable to COVID-19 according to UK governmental guidance and 1,208 (60%) did not.
Recruitment	Recruitment criteria into the Virus Watch study were residence in England or Wales, household size up to six people with consent or assent of all household members, access to an email address, and ability to complete English-language surveys. The source population were households from the general population across all national regions of England and Wales. Households were recruited from the general population using several methods, including postal recruitment using a probability sample derived from the Royal Mail Post Office Address File, as well as including letter and SMS-based recruitment supported by general practices, and social media campaigns (aiming to reach a diverse population). Participants were from the Virus Watch study and met the inclusion criteria stipulated in 'Field Specific Reporting'. Further detail is provided in the Methods section.
Ethics oversight	Approved by the Hampstead NHS Health Research Authority Ethics Committee: 20/HRA/2320, and conformed to the ethical standards set out in the Declaration of Helsinki. All participants provided informed consent for all aspects of the study.

Note that full information on the approval of the study protocol must also be provided in the manuscript.

Field-specific reporting

Please select the one below that is the best fit for your research. If you are not sure, read the appropriate sections before making your selection.

☒ Life sciences ☐ Behavioural & social sciences ☐ Ecological, evolutionary & environmental sciences

For a reference copy of the document with all sections, see nature.com/documents/nr-reporting-summary-flat.pdf

Life sciences study design

All studies must disclose on these points even when the disclosure is negative.

Sample size	N=2010, a adult (>18 year old) sub-cohort from the Virus Watch prospective cohort study who met the following inclusion criteria required to be eligible for the analyses:<br>1) participated in the serological sub-cohort of the study and returned at least one finger-prick antibody sample with a valid result for anti-S and/or anti-N antibodies,<br>2) completed survey(s) about new-onset long-term symptoms which covered symptom development between February 2020 and March 2023, and had binary classifiable Post Covid Condition status,<br>3) had their first recorded SARS-CoV-2 infection detected via polymerase chain reaction (PCR) or lateral flow test (LFT) before end of serological follow-up,<br>4) mild-moderate infection (i.e., convalesced in the community without hospitalisation).<br><br>Further detail is provided in the Methods section.
Data exclusions	The pre-specified exclusion criteria were not meeting the inclusion criteria stipulated above and were determined by the focus of the study. This study focused on primary infections, and consequently post-infection follow-up was limited to primary infections (i.e. not reinfections). All available data for participants who met the inclusion criteria were included in the study. Vaccination-related serological data were limited to doses up to the second dose due to the timing of the study and consequent data availability. Further detail provided in the Methods section.
Replication	This was an original prospective cohort study and consequently there was no experimental design to be replicated. We have provided specific methodological detail of all phases of the study to enable replication in future cohorts in the Methods section.
Randomization	This was not an experimental study. Covariates were controlled using adjustment and stratification, described in detail in the Methods section.
Blinding	N/A as this was not an experimental study.

Reporting for specific materials, systems and methods

We require information from authors about some types of materials, experimental systems and methods used in many studies. Here, indicate whether each material, system or method listed is relevant to your study. If you are not sure if a list item applies to your research, read the appropriate section before selecting a response.

Materials & experimental systems

n/a	Involved in the study	
☐	☒	Antibodies
☒	☐	Eukaryotic cell lines
☒	☐	Palaeontology and archaeology
☒	☐	Animals and other organisms
☒	☐	Clinical data
☒	☐	Dual use research of concern
☒	☐	Plants

Methods

n/a	Involved in the study	
☒	☐	ChIP-seq
☒	☐	Flow cytometry
☒	☐	MRI-based neuroimaging

Antibodies

Antibodies used	Roche Elecsys Anti-SARS-CoV-2 nucleocapsid (N) and spike (see Methods section)
Validation	Described in Methods section ('Outcomes' subsection) including published validation literature

Plants

Seed stocks	*Report on the source of all seed stocks or other plant material used. If applicable, state the seed stock centre and catalogue number. If plant specimens were collected from the field, describe the collection location, date and sampling procedures.*
Novel plant genotypes	*Describe the methods by which all novel plant genotypes were produced. This includes those generated by transgenic approaches, gene editing, chemical/radiation-based mutagenesis and hybridization. For transgenic lines, describe the transformation method, the number of independent lines analyzed and the generation upon which experiments were performed. For gene-edited lines, describe the editor used, the endogenous sequence targeted for editing, the targeting guide RNA sequence (if applicable) and how the editor was applied.*
Authentication	*Describe any authentication procedures for each seed stock used or novel genotype generated. Describe any experiments used to assess the effect of a mutation and, where applicable, how potential secondary effects (e.g. second site T-DNA insertions, mosiacism, off-target gene editing) were examined.*
